# Prediction of vancomycin initial dosage using artificial intelligence models applying ensemble strategy

**DOI:** 10.1186/s12859-022-05117-8

**Published:** 2023-03-22

**Authors:** Wen-Hsien Ho, Tian-Hsiang Huang, Yenming J. Chen, Lang-Yin Zeng, Fen-Fen Liao, Yeong-Cheng Liou

**Affiliations:** 1 Department of Healthcare Administration and Medical Informatics, Kaohsiung Medical University, No. 100, Shin-Chuan 1st Road, Kaohsiung, 807 Taiwan; 2 Department of Medical Research, Kaohsiung Medical University Hospital, No. 100, Shin-Chuan 1st Road, Kaohsiung, 807 Taiwan; 3 College of Professional Studied, National Pingtung University of Science and Technology, No. 1, Shuefu Road, Pingtung, 912 Taiwan; 4 Department of Computer Science and Information Engineering, National Penghu University of Science and Technology, No.300, Liuhe Road, Magong, 880 Penghu Taiwan; 5 Department of Information Management, National Kaohsiung University of Science and Technology, No.1, University Road, Kaohsiung, 824 Taiwan; 6 Department of Pharmacy, Kaohsiung Medical University Hospital, No. 100, Shin-Chuan 1st Road, Kaohsiung, 807 Taiwan; 7 Research Center of Nonlinear Analysis and Optimization, Kaohsiung Medical University, No. 100, Shin-Chuan 1st Road, Kaohsiung, 807 Taiwan

**Keywords:** Vancomycin, Ensemble strategy, Monitoring of blood concentration of drugs, Therapeutic drug monitoring (TDM)

## Abstract

**Background:**

Antibiotic resistance has become a global concern. Vancomycin is known as the last line of antibiotics, but its treatment index is narrow. Therefore, clinical dosing decisions must be made with the utmost care; such decisions are said to be “suitable” only when both “efficacy” and “safety” are considered. This study presents a model, namely the “ensemble strategy model,” to predict the suitability of vancomycin regimens. The experimental data consisted of 2141 “suitable” and “unsuitable” patients tagged with a vancomycin regimen, including six diagnostic input attributes (sex, age, weight, serum creatinine, dosing interval, and total daily dose), and the dataset was normalized into a training dataset, a validation dataset, and a test dataset. AdaBoost.M1, Bagging, fastAdaboost, Neyman–Pearson, and Stacking were used for model training. The “ensemble strategy concept” was then used to arrive at the final decision by voting to build a model for predicting the suitability of vancomycin treatment regimens.

**Results:**

The results of the tenfold cross-validation showed that the average accuracy of the proposed “ensemble strategy model” was 86.51% with a standard deviation of 0.006, and it was robust. In addition, the experimental results of the test dataset revealed that the accuracy, sensitivity, and specificity of the proposed method were 87.54%, 89.25%, and 85.19%, respectively. The accuracy of the five algorithms ranged from 81 to 86%, the sensitivity from 81 to 92%, and the specificity from 77 to 88%. Thus, the experimental results suggest that the model proposed in this study has high accuracy, high sensitivity, and high specificity.

**Conclusions:**

The “ensemble strategy model” can be used as a reference for the determination of vancomycin doses in clinical treatment.

## Background

Antimicrobial resistance (AMR) has become a major global concern. The World Economic Forum has stated that “arguably the greatest risk… to human health comes in the form of antibiotic-resistant bacteria.” [1]. In 2014, the World Health Organization conducted a global study on drug resistance with data from 114 countries, which confirmed that drug resistance is a global crisis [2]. According to recent statistics, by 2050, drug-resistant infectious diseases will kill more people than cancers do [3]. In particular, groups with a high risk of infection, such as elderly patients and those with cancer, require high doses of antibiotics and prolonged treatment, which lead to antibiotic-resistance.

Antibiotics are being developed at a much slower rate than the growth rate of drug-resistant bacteria. The number of antimicrobial agents approved for marketing by the U.S. Food and Drug Administration has been declining from 1983 to 2011. No new antimicrobial agents have been introduced in the last 20 years. Therefore, there are likely to be no antimicrobial agents with new mechanisms available for clinical use for a long period. Consequently, it is essential to adopt appropriate dose control of antimicrobial agents for medical and animal use.

Vancomycin is currently classified as a third-line antibiotic by the Ministry of Health and Welfare in Taiwan, and the higher the level, the greater the risk. Vancomycin is often used to treat severe infections in which all other antibiotics are ineffective and is also known as the “last line of drugs.” The use of vancomycin is strictly limited. Thus, it is a serious problem if patients develop resistance to the drug. Moreover, owing to the narrow treatment index of vancomycin, there is a risk of toxicity, and patients may develop adverse effects such as nephrotoxicity and ototoxicity [4], allergic reactions, renal impairment, or even cardiac arrest [5].

There are many factors affecting the administration of vancomycin with individual variations, such as the renal function, body condition, and hypoproteinemia of the patient [6]. Most patients receiving antibiotics already have serious infections, and the renal damage caused by the drug or a wrong drug administration decision may promote drug resistance. The daily dose and dose interval of vancomycin as well as the use of therapeutic drug monitoring (TDM) in combination with individualized clinical dosing are extremely important.

Trough concentrations are commonly used in the indicator monitoring of vancomycin. To alleviate the risk of nephrotoxicity, it is best to maintain the trough concentration of vancomycin between 10 and 20 mg/L [7]. In the past, clinicians relied on nomograms [8] or pharmacokinetics for dose adjustment in vancomycin therapy [9], but these methods may not be effective. Therefore, in 2007, Hu employed the decision tree induction algorithm C4.5 and back-propagation network to construct a decision support system (i.e., trough and peak concentrations) [4].

However, it is more important for the clinicians to predict the suitability of the dose instructions given in a treatment regimen than to predict the blood drug concentrations—the trough and peak concentrations. Therefore, in recent years, studies have leaned toward predicting the suitability of vancomycin treatment regimens, of which, several investigations have used group pharmacokinetic software. For example, Xu et al. [10] used dose calculation to predict the trough concentrations for obtaining stability, while Nunn et al. [11] used studies to verify the accuracy of the software. In addition, Leu et al. [12] used a conventional nomogram for dosing to control the trough concentration of vancomycin. Finally, Xu et al. [10] studied past data using subgroups with and without pharmacist intervention to ascertain the presence of differences.

Since 1990, after Schapire [13] proposed Bayesian Averaging Ensemble Learning, the research on ensemble learning has been gaining attention. The concept is to construct a model by combining multiple learning algorithms. Such a model usually has superior predictive power than the individual algorithms. The approach is called ensemble learning because it is mostly a combination of basic learning algorithms. However, few studies have used this strategy to predict the suitability of vancomycin decision regimens. Only Hu et al. [4] used decision trees and bagging to build a model; however, the accuracy rate was only about 60%. Ho et al. [14] proposed the use of genetic algorithms and improved the Taguchi algorithm to predict the suitability of the vancomycin decision, which had an accuracy rate of 87.5%. In this study, the ensemble strategy was used to model five ensemble learning algorithms, and the results were filtered by voting.

The patient data of this study, which contained six input variables and one output variable, were trained using the package R-STUDIO version 3.6.0 to implement five algorithms: AdaBoost.M1 [15], Bagging [16], fastAdaboost, Neyman–Pearson [17] and Stacking [18, 19]. The results of these five algorithms were filtered using the majority rule to establish an ensemble strategy to predict the suitability of the initial dosing decision for vancomycin. The results indicated that this approach outperformed the previous studies in terms of the measurement indicators. In addition, with the shortened duration of treatment and reduced unnecessary risks, it further helped clinicians evaluate the initial dose more carefully to enhance the safety and efficacy of drug administration.

## Results and discussion

In this study, an ensemble strategy was proposed to predict the suitability of the vancomycin dosing regimen. The experimental results containing three measurement indicators—accuracy, sensitivity, and specificity—are shown in Table 1.

**Table 1:** Experimental results of the three measurement indicators

Algorithm	Dataset	Accuracy (%)	Sensitivity (%)	Specificity (%)
AdaBoost.M1	Training	95.33	96.64	93.06
	Validation	87.64	88.78	86.31
	Test	87.23	92.47	80.00
Bagging	Training	84.27	88.95	77.68
	Validation	81.04	87.76	73.21
	Test	81.93	87.63	74.07
fastAdaboost	Training	99.86	100.0	99.67
	Validation	89.84	88.78	91.07
	Test	84.11	89.25	77.03
Neyman–Pearson	Training	97.18	99.29	94.21
	Validation	89.56	88.78	90.48
	Test	84.73	81.72	88.89
Stacking	Training	99.86	100.0	99.67
	Validation	91.76	90.31	93.45
	Test	86.29	88.71	82.96
Ensemble strategy	Test	87.54	89.25	85.19

The experimental results demonstrated that the accuracy of the testing set models of the five algorithms ranged from 81.93 to 87.23%. Furthermore, the accuracy of the rebuilt models after filtering by voting increased to 87.54%. The sensitivity was second only to the AdaBoosting.M1 algorithm, and the specificity was second only to the Stacking algorithm. The confusion matrix in Table 2 further suggested that the false-positive rate was only 12%. As this study was aimed at helping the clinicians to predict the suitability of vancomycin dosing decisions, it is desirable to have a low false-positivity rate.

**Table 2:** Confusion matrix of the ensemble strategy model

		Actual values	
		Suitable	Unsuitable
Model predicted values	Suitable	115	20
	Unsuitable	20	166

**Fig. 1:**
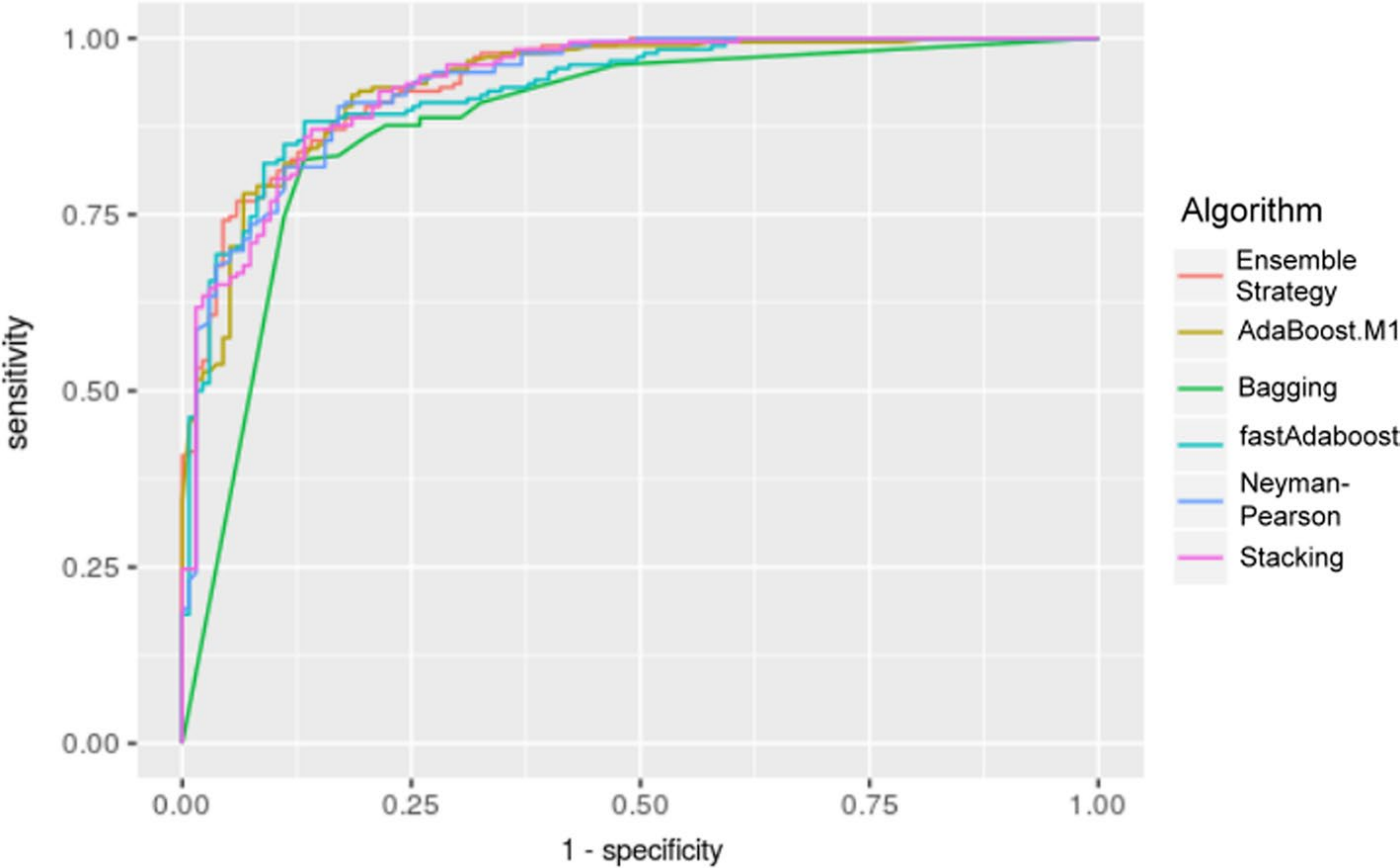
Receiver operator characteristic curve: the ROC curve of our proposed ensemble strategy converges most smoothly

The results of the measurement indicators signified that the ensemble strategy proposed in this study had a good performance. The ROC curve (Fig. 1) and the AUC values (Table 3) are as follows:

**Table 3:** Area under the ROC curve (AUC) values

Method	AUC value
Ensemble strategy	0.940
AdaBoost.M1	0.936
Bagging	0.881
fastAdaboost	0.928
Neyman–Pearson	0.935
Stacking	0.936

The data in Table 5 show that the ensemble strategy has the highest AUC value, which is close to 1.0, indicating the authenticity of this detection to an extent.

Antibiotics are being developed slowly, but high doses of antibiotics and prolonged treatment often lead to antibiotic-resistance, then to kill more and more people. Therefore, it is essential to adopt appropriate dose control of antimicrobial agents for medical use. Vancomycin is known as the "last line of drugs" in Taiwan. The daily dose and dose interval of vancomycin are extremely important. Some studies tried to predict the suitability of the dose instructions given in a treatment regimen. But most results' accuracies are not good, except Ho et al. [14]. In this study, the ensemble strategy was proposed to predict the suitability of the vancomycin decision by voting five ensemble learning algorithms. The experimental results show that our proposed strategy has the highest accuracy and is robust, while taking into account the advantages of high sensitivity and high specificity, in addition with low false positives and the highest AUC value.

## Conclusions

The model developed in this study is expected to assist physicians in initial dosing decisions and serve as a reference tool in clinical decision-making. As vancomycin is often administered to patients after the initial dose and then adjusted based on the trough concentration of the drug in the subsequent blood test report, the ensemble strategy is expected to enhance the safety and effectiveness of the dosing decisions. Therefore, if the initial dosing decision is optimized, the treatment duration can be shortened and the risk of drug resistance and toxicity can be reduced.

The experimental results have showed that the model proposed in this study has the highest accuracy with excellent sensitivity, specificity, and low false-positivity, which proved that the ensemble strategy approach recommended in this study is worth adopting.

## Methods

### Implementing five algorithms

In this study, the “adabag” package of R-STUDIO version 3.6.0 was used to implement the AdaBoost.M1 and Bagging algorithms, the “fastAdaboost” package to implement the fastAdaboost algorithm, the “nproc” package to implement the Neymain-Pearson algorithm, and the “SuperLearner” package to implement the Stacking algorithm. Bagging used a classification tree as a single classifier; the iterated logarithm (mfinal) of fastAdaboost was set to the best possible 50 iterations; Neyman–Pearson used Random Forest as the basic classification method, and the acceptable statistical Type I error was set to 0.05; Stacking also used Random Forest as the basic classification method, and the family parameter was set to Binomial.

**Fig. 2:**
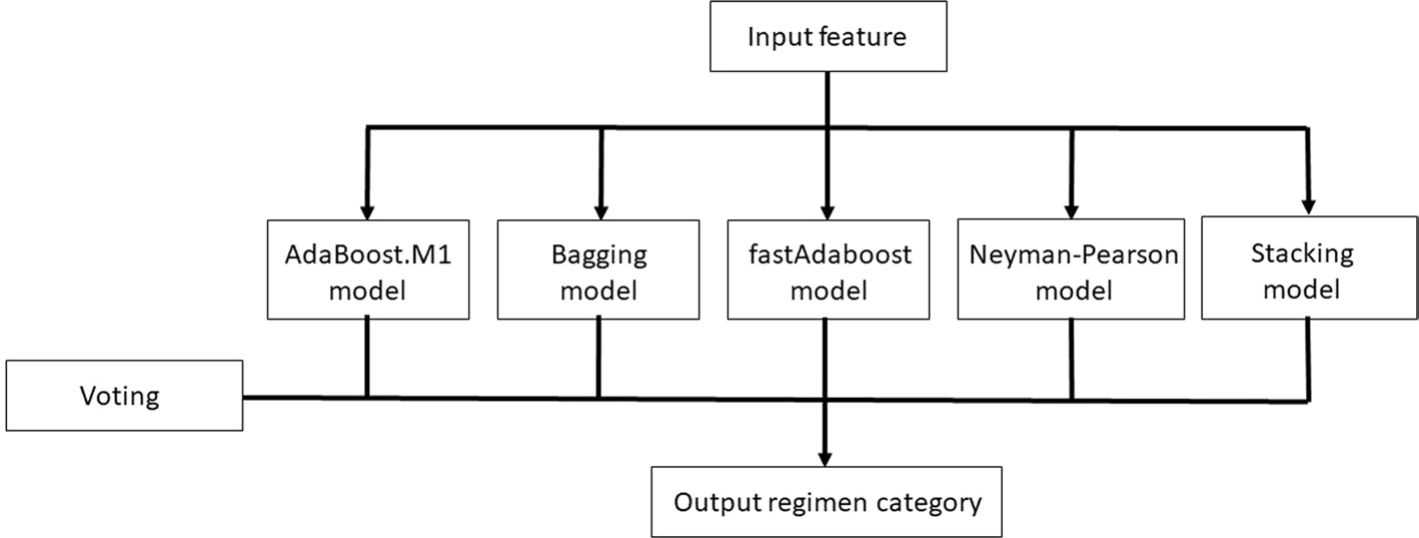
The architecture of the “ensemble strategy concept”: 6 features were input to 5 machine learning algorithms, then the output regimen category label was predicted by the voting scheme

The “ensemble strategy concept” was used to predict the suitability of the vancomycin decision after filtering by (majority rule) voting above-mentioned five ensemble learning algorithms, including AdaBoost.M1, Bagging, Boosting, fastAdaboost, Neymain-Pearson and Stacking algorithms. Figure 2 depicts the architecture of the “ensemble strategy concept.” The input features are gender, age, weight, serum creatinine (SCR), dosing interval, total daily dose of medication, and one output regimen category label to indicate if suitable vancomycin dosing or not.

### Measurement indicators

To understand the generalization ability of the model, three performance measurement indicators were used, namely, accuracy, sensitivity (also known as true positive and recall), and specificity, which were calculated using Eqs. (1–3). In addition, the receiver operating characteristic (ROC) curve was plotted, and the area under the ROC curve (AUC) values were calculated to understand the model optimization via the value of the indicators.

1 $${\text{Accuracy}} = \frac{{{\text{TP}} + {\text{TN}}}}{{{\text{TP}} + {\text{FP}} + {\text{FN}} + {\text{TN}}}},$$

2 $${\text{Sensitivity}} = \frac{{{\text{TP}}}}{{{\text{TP}} + {\text{FN}}}},$$

3 $${\text{Specificity}} = \frac{{{\text{TN}}}}{{{\text{FP}} + {\text{TN}}}},$$

where TP stands for true positive, which is predicted to be suitable and actually suitable; TN stands for true negative, which is predicted to be unsuitable and actually unsuitable; FP stands for false positive, which is predicted to be suitable but actually unsuitable, and is also a statistical Type I error; FN stands for false negative, which is predicted to be unsuitable but actually suitable and is also a statistical Type II error. Thus, accuracy represents the number of people who were correctly determined as suitable out of all decisions; sensitivity is the percentage of decisions that were successfully predicted to be suitable in cases where they were actually suitable; and specificity is the percentage of decisions that were successfully predicted to be unsuitable in cases where they were actually unsuitable.

The ROC curve is an analytical tool with coordinate plots and is the simplest and most intuitive observation method to analyze clinical accuracy. The plot can be used to make direct judgments from the curves [20]. The vertical coordinates denote the true positive rate (sensitivity) and the horizontal coordinates denote the false-positive rate (1-specificity), reflecting the relationship between the specificity and sensitivity of an analytical method. The diagonal line is the reference line. If the ROC curve, as the testing tool, is located exactly on the diagonal reference line, it means that the testing tool is not discriminative in terms of the prediction. If the ROC curve moves to the upper left, the tool is more sensitive to prediction and the false-positive rate is lower, i.e., the tool has better discriminative power. The point closest to the upper left corner (0, 1) is the cutoff point with the least misclassification, where the sensitivity is the largest, and the false-positive rate (1-specificity) is the smallest [21, 22].

The AUC is the area under the ROC curve, and the value usually ranges from 0.5 to 1. Thus, the higher the AUC value, the better it is, and the closer it is to 1.0, the higher its truthfulness [23].

### Datasets

The data collection period was from 2011 to 2018; 2141 data were collected, including factors related to antibiotic dose decisions (variables), as shown in Table 4. The selection of these variables was the same as those in studies performed by Hu et al. [4] and Ho et al. [14]. The database was reviewed and approved by the Human Investigation Committee of the Kaohsiung Medical University Hospital (KMHIRB-E(I)-20190364). The inclusion criteria were: the patients were hospitalized at the hospital of the Kaohsiung Medical University system, and the hospitalization report matched one of the following vancomycin health insurance drug codes: B018156277, AC41443277, A041443277, AC37290277, AC575743277, AC37290277, AC37290277, AC37290277, AC57430277, AC5743277. AC37290277, AC57286277, BC17742277, and BB17742277.

**Table 4:** Definition of operating variables

Variables	Type	Range
Gender	Category	Male/female
Age	Continuous	14–93 (age)
Weight	Continuous	34–100 (kg)
Serum creatinine (SCR)	Continuous	0.25–5.90
Dosing interval	Category	6–24 (hours)
Total daily dose of medication	Continuous	125–4000 (mg)
Suitability of vancomycin dosing regimen	Category	Suitable/unsuitable

The recommended doses of antimicrobial agents have been mostly studied in younger age groups. However, the recommended doses for the elderly, newborns, and children should be adjusted [7], especially since the vancomycin dose for children is far different from that for adults. The latest version of the revised consensus on methicillin-resistant *Staphylococcus aureus* infection also pointed out the differences in doses for children and adults [7]. Thus, the present study excluded infants from the study population.

Since obese patients have more fat distribution, the dose calculation should be evaluated carefully based on the real bodyweight or the corrected body weight according to the lipophilicity of the drug. In addition, clinicians usually determine the initial dose based on the patient's infection status, body weight, and renal function. The dose is calculated based on the patient's body weight for drugs with a narrow treatment range. Therefore, body weight was one of the important factors examined in this study.

Serum creatinine (SCR) is derived from the decomposition of serum creatinine due to normal muscle activity. SCR would be filtered from the blood excreted with urine in people having normal kidney functions, where the kidney is responsible for more than 90% of creatinine metabolism. As a result, SCR can be used as an indicator to monitor kidney functions. The normal value of SCR varies with gender. Thus, gender was also included as an input value.

Ultimately, the dependent category variable of Table 4—“suitability of vancomycin dosing regimen” is classified according to the value of trough-based vancomycin TDM. The trough concentration of the drug in the blood test report is better between 10 and 20 mg/L [7].

### Descriptive statistics of the datasets

The descriptive statistics about the datasets are shown in Table 5.

**Table 5:** Descriptive statistics of the dataset

Variables	Overall (n = 2141)
*Gender (%)*	
Male	1300 (60.72%)
Female	841 (39.28%)
Age	63.75 ± 16.23
Bodyweight	61.70 ± 12.07
Serum creatinine (SCR)	1.30 ± 1.06
*Dosing interval (%)*	
6 h	195 (9.10%)
8 h	274 (12.80%)
12 h	857 (40.03%)
24 h	815 (38.07%)
Total daily dose	1387.05 ± 1006.31
*Suitability of vancomycin dosing regimen (%)*	
Suitable	916 (42.78%)
Unsuitable	1225 (57.22%)

### Pre-processing and partitioning of data

As the differences in the units of data may affect the results, this study scaled the data equal to the interval of 0–1 using the Min–Max scaling formula and thus improved the rate of convergence and model accuracy [24].

4 $$X_{nom} = \frac{{X - X_{min} }}{{X_{max} - X_{min} }},$$

where $X_{nom}$ represents the result of data normalization, $X_{min}$ represents the minimum value in the data, and $X_{max}$ represents the maximum value.

After pre-processing, 85% of the data were randomly partitioned for model training, where 80% were used as the training dataset, 20% as the validation dataset, and the remaining 15% as the test dataset to validate the model. To ensure the evaluation of the model fitting performance, tenfold cross-validation was used in this study.

## Data Availability

The data that support the findings of this study are available from Kaohsiung Medical University Hospital but restrictions apply to the availability of these data, which were used under license for the current study, and so are not publicly available. Data are however available from the authors upon reasonable request and with permission of Kaohsiung Medical University Hospital.

## Abbreviations

AMR: Antimicrobial resistance
TDM: Therapeutic drug monitoring
ROC: Receiver operating characteristic
AUC: Area under the ROC curve
SCR: Serum creatinine
